# Hazardous alcohol consumption is not associated with CD4+ T-cell count decline among PLHIV in Kampala Uganda: A prospective cohort study

**DOI:** 10.1371/journal.pone.0180015

**Published:** 2017-06-30

**Authors:** Bonnie Wandera, Nazarius M. Tumwesigye, Joaniter I. Nankabirwa, Andrew D. Kambugu, David K. Mafigiri, Saidi Kapiga, Ajay K. Sethi

**Affiliations:** 1 Department of Epidemiology and Biostatistics, Makerere University School of Public Health, Kampala, Uganda; 2 Infectious Diseases Institute, Makerere University College of Health Sciences, Kampala, Uganda; 3 Department of Medicine, Makerere University College of Health Sciences, Kampala, Uganda; 4 Department of Social Work and Social administration, Makerere University College of Humanities and Social Sciences, Kampala, Uganda; 5 Department of Infectious Disease Epidemiology, London School of Hygiene & Tropical Medicine and Mwanza Interventional Trials Unit, Mwanza, Tanzania; 6 Department of Population Health Sciences, University of Wisconsin-Madison, Madison, Wisconsin, United States of America; Stellenbosch University, SOUTH AFRICA

## Abstract

**Introduction:**

There is limited data on the effects of alcohol on immunological response among persons living with HIV (PLHIV) in sub-Saharan Africa. We assessed the relationship between hazardous alcohol use and CD4+ T-cell count, among PLHIV in Uganda.

**Methods:**

PLHIV aged ≥ 18 years were enrolled in a cohort study at the Infectious diseases clinic Kampala, Uganda. Alcohol consumption was assessed at enrolment (baseline) and 6 monthly thereafter using the alcohol use disorders identification test (AUDIT). The CD4+ T-cell counts, assessed at baseline and over the next 12 months were compared between alcohol use strata, using linear mixed effects regression. Using longitudinal mediation analysis methods, we estimated the effect of alcohol induced ART non-adherence on CD4+ T-cell count.

**Results:**

Of the 1566 participants enrolled, 863(44.1%) were non-alcohol users (AUDIT score = 0), 433(27.7%) were non-hazardous (AUDIT score 1–7) alcohol users while 270 (17.2%) were hazardous (AUDIT score ≥ 8) alcohol users.

The overall median (IQR) baseline CD4+ T-cell count was 356 (243–516) cells/μl. There were no differences in the median baseline CD4+ T-cell count between hazardous and non-hazardous alcohol users compared to non-alcohol users in both the non-ART (p = 0.43) and ART group (p = 0.77).

The mean CD4+ T-cell count over 12 months was not different between hazardous alcohol users and non-alcohol users (non-ART group p = 0.88 and ART group p = 0.62), nor between non-hazardous alcohol users and non-alcohol users (and non-ART group p = 0.66 and ART group p = 0.20).

Alcohol use was not associated with a significant natural direct effect on CD4^+^ T-cell count (1.37 95%CI [-1.78, 4.52] cells/μl, p = 0.39) but had a statistically significant natural indirect effect on reduction of CD4^+^ T-cell count (-0.91 cells/μl [-1.36, -0.45], p < 0.001) mediated through ART non-adherence.

**Conclusion:**

Hazardous alcohol use among PLHIV was not directly associated with lower CD4+ T-cell count but had a significant natural indirect effect on CD4+ T-cell count mediated through ART non-adherence. Among PLHIV with lower than expected CD4+ T-cell count, alcohol consumption should be excluded as an underlying factor for non-adherence to ART and any interventions targeting alcohol use should tackle possible ART non-adherence.

## Introduction

Alcohol consumption is common among persons living with HIV (PLHIV) both globally and in sub-Saharan Africa (SSA) [1,2]. Consumption of alcohol may result in deleterious effects on the host immune defenses through promoting pro-inflammatory immune responses, impairing anti-inflammatory cytokines, accelerating T-cell apoptosis as well as mitochondrial dysregulation [3]. Chronic administration of alcohol in an SIV infected -macaque model showed depletion of circulating host CD4+ T-cells [4]. Alcohol use has also been shown to delay seeking HIV care, result in poor adherence to antiretroviral therapy (ART) as well as increase the likelihood of dropping out of HIV care[5–8]. Taken together, these biological mechanisms directly and the behavioral mechanisms indirectly may lead to increased HIV viral replication and CD4+ T-cell count decline among untreated PLHIV, as well as impaired CD4+ T-cell count reconstitution among PLHIV on ART[9].

Some studies that explored the clinical impact of alcohol use on the immunological responses in PLHIV demonstrated a significant association while others did not demonstrate any association. For example, among persons not yet on ART, the multi-center AIDS cohort study, consisting of men who have sex with men and another study of HIV infected heterosexual men and women not on ART in Zimbabwe [10,11] as well as a longitudinal study among 266 women not on ART in the HIV Epidemiologic Research Study (HERS) cohort showed no significant association between level of alcohol use and CD4+ T-cell count[12].

However, a 7 year longitudinal study in the United States of America showed that participants with heavy alcohol use who were not yet on ART had on average 49 CD4+ T-cells/μl lower compared to abstinent participants[13].

Among participants using ART, the results are again mixed with 3 longitudinal studies from the USA and one from the Swiss HIV cohort demonstrating no association between heavy alcohol consumption and having lower CD4+ T-cell counts[13–16] while *Baum et al* demonstrated that frequent alcohol use significantly increased the hazard of CD4^+^ cell count decline to ≤ 200 cells/μl over 30 months of follow-up compared to non-alcohol use[17]. From available studies, mainly done in Europe and North America, there is no consensus on whether consumption of alcohol results in a reduction in CD4+ T-cell counts among participants in clinical settings. A cross-sectional study from South Africa showed that PLHIV with hazardous/harmful alcohol use were significantly more likely to have lower CD4+ T-cell counts than alcohol abstinent participants [18]. However, the cross-sectional design of the latter study limits the strength of evidence because of the concurrent assessment of both alcohol use and CD4+ T-cell count.

Generalizing these findings to SSA may not be appropriate because of the different prevalent HIV subtypes in this region, which inherently have different HIV disease progression rates [19,20]. Some HIV treatment programs in Uganda continue to use CD4+ T-cell count (in the absence of plasma viral load tests) as an objective marker of ART response and monitoring [21].

The above differences, the dearth of similarly appropriate studies from SSA, in the setting of the wide availability and use of alcohol in SSA, justifies locally appropriate studies to assess the effect of alcohol on HIV disease progression in this setting. Exploring the association of alcohol use with CD4+ T-cell count among PLHIV in this setting has major implications on the HIV/AIDS care, treatment and ART monitoring in this setting as well as contribution to the global unanswered question of the effect of alcohol use on CD4+ T-cell count[9].

Therefore, we assessed whether hazardous alcohol use is associated with lower CD4+ T-cell count over 12 months among HIV infected men and women with and without ART in an urban HIV clinic in Kampala Uganda. Furthermore, we examined whether effects of alcohol consumption on CD4+ T-cell count among persons on ART were indirectly mediated through reduction in ART adherence.

## Methods

### Study design, setting and sampling

PLHIV at the adult Infectious Diseases Institute (IDI) clinic located within the Mulago National Referral and Teaching Hospital Kampala City, were systematically screened for enrollment into this prospective alcohol cohort study. Details of the clinic services and population have been previously described [22].

Between October 2012 and May 2014, all patients presenting to the clinic were registered upon arrival. On every clinic day, we sampled the 15^th^subject to arrive and thereafter every 15th patient in the clinic arrival sequence and assessed their study eligibility. Patients were enrolled if they were i) aged ≥18 years; ii) healthy enough to answer interview questions as assessed by a Karnofsky clinical performance score >50%;iii) willing to provide written informed consent for study participation; and iv) self-reported as not pregnant, if female and v) willing to continue receiving care at the clinic for the next 12 months.

### Data collection procedures

#### Baseline data collection

At study enrollment, a structured questionnaire was administered by trained nurse counselors in English or Luganda (a local language). Demographic characteristics; age, gender, religion, employment status, participants’ income in the last month and highest educational level attained were collected.

Alcohol consumption within the last 6 months was ascertained through interview using the AUDIT questionnaire and additional information on actual days on which alcohol consumption occurred within the past 30 days and “usual” amounts and type of alcohol consumed was collected using the Alcohol Timeline Follow-back method[23,24].

Adherence to prescribed ART (or co-trimoxazole for those not yet on ART) in the last month was assessed on self-reported ranking on a visual analogue scale (VAS) of all antiretroviral drugs (or co-trimoxazole) taken out of all antiretroviral drugs (or co-trimoxazole) prescribed within the last month. All participants receive co-trimoxazole (or dapsone) for prophylaxis against respiratory and diarrheal infections that is taken daily even after initiating ART.

Depression was assessed using the 10-item Center for Epidemiology Studies on Depression tool (CESD-10). The CES-D has been validated in sub-Saharan Africa [25]. Participants were considered depressed if they scored at least 10 points on the CESD-10.

Clinical data, abstracted from the participants’ records included: date of first HIV positive test result, WHO HIV/AIDS disease stage, ART receipt status, as well as every 6 months (+/- 3 months) absolute CD4+ T-cell count and CD4 percent.

#### Follow-up data collection

Over the next 12 months after the enrollment, we abstracted the participants’ data from clinic records. Follow-up data obtained was; absolute CD4+ T-cell count, CD4 percent and self-reported adherence to ART or co-trimoxazole for non-ART participants. Although as per clinic protocols, every participant is ordinarily scheduled to have a repeat CD4+ T-cell count every 6 months, not all participants had the same number of CD4 tests done. All CD4+ T-cell count and CD4 percentage tests were done at a college of American Pathologists certified Makerere-University John Hopkins University laboratory using FACSCalibur (Becton Dickinson, San Jose, CA).

### Data management and analysis

All data were double entered in a customized Microsoft Access (Redmond, WA, USA) database and exported to SAS V 9.1.3 (SAS Inst., Carry, NC, USA) for statistical analysis.

All tests were 2 sided with significance level set at 0.05. All analyses were stratified by ART receipt strata.

The exposure of interest, hazardous alcohol use, was defined as scoring ≥ 8 points on the AUDIT, while participants who scored between 1–7 points were classified as having non-hazardous alcohol use[26,27]. Participants who reported no alcohol use (AUDIT score = 0) in the last 6 months were classified as non-alcohol users.

The outcomes of interest were, differences in the i) median CD4+ T-cell count and CD4 percent at the enrollment visit and ii) mean CD4+ T-cell count and CD4 percent, within the 3 alcohol use strata over the 12 months after the alcohol assessment interview.

Continuous variables (CD4+ T-cell count, age, and CD4 percent) were summarized as means with standard deviations (SD) or medians with inter-quartile ranges (IQR) while categorical variables were summarized as percentages.

The median CD4+ T-cell count and CD4 percent were compared between the three alcohol use strata (hazardous, non-hazardous and non- alcohol use) using the Kruskal- Wallis test.

We built a multivariable linear mixed effects (LME) regression model to assess the difference in mean CD4+ T-cell count by alcohol use strata while simultaneously accounting for potential confounding factors. The LME model was based on restricted maximum likelihood estimation, with a random intercept, random slope and an unstructured covariance matrix with CD4+ T-cell count as the outcome and alcohol use strata as the exposure. The LME model adjusts for the correlations of repeated CD4+ T-cell count values within the same participant, allowing them to change with time between their measurements as well as handle the differing number of visits per participant[28].

The potential confounders of the CD4+ T-cell count and alcohol use relationship identified from prior research or those with p-values ≤0.2 from univariable analyses were selected for the initial multivariable model. The modelling process involved the iterative removal of the factor with the highest non-significant p-value from the multivariable model if its removal didn’t result in>10% change in the parameter estimate of the alcohol use variable. This process was iteratively repeated until there was no additional factor eligible for removal to arrive at the final multivariable model. We reviewed the histogram plotted of the distribution of random intercepts and slopes and found them acceptably normally distributed.

The above modelling processes were repeated with CD4+ T-cell percent as the outcome of interest.

Furthermore, using longitudinal mediation analysis methods, we assessed whether the effects of hazardous alcohol consumption on CD4+ T-cell count are mediated through ART adherence. We estimated the natural direct effect and natural indirect effect of alcohol use on CD4+ T-cell count among persons receiving on ART using structural equation models, with bootstrap (reps = 1000) estimation of standard errors. In this part of the analysis, alcohol use was analysed as an ordinal variable of 3 levels from non-alcohol user as the lowest level, to non-hazardous alcohol users and hazardous alcohol users as the highest on the scale.

### Ethics statement

The study was approved by the scientific and ethical review boards of IDI, Makerere University School of Public Health Research and Ethics Committee and the Uganda National Council of Science and Technology.

## Results

### Descriptive characteristics

Between 4^th^ December 2013 and 4^th^ October 2014 we approached 1618 participants of whom 1566 participants were enrolled. Of the 52 subjects not enrolled, 35 (67.3%) were females. Reasons for non-enrollment were: no time for study participation (n = 17/52), not interested (n = 16/52), understands neither interview languages (n = 3/52), 2 were pregnant, while 14/52 participants were not enrolled due to other reasons.

Females constituted 54% (n = 846) of the study population while the mean (SD) age was 39.0 (10.0) years. The majority, 900 (57.8%) were in WHO HIV disease stage III & IV, with median (IQR) CD4+ T-cell count of 370 (257–519) while 83.3% (1304) were receiving ART. Descriptive characteristics of the study population by alcohol use strata are presented in Table 1.

**Table 1:** Baseline descriptive characteristics of 1566 PLHIV, stratified by alcohol use, category enrolled in the alcohol study at the Infectious Diseases Institute, Kampala Uganda.

	No alcohol use reported in last 6 months N = 863	Non-hazardous Alcohol use within last 6 months N = 433	Hazardous Alcohol use in last 6 months N = 270	Total participants N = 1566
Mean age in years, (SD)	38.4(9.0)	39.0(10.1)	39.6(10.2)	39.1(10.0)
Duration years with HIV, median (IQR)	6.7(3.9–8.5)	6.0(3.7–8.4)	4.8(2.9–7.9)	6.3(3.6–8.4)
HIV/AIDS clinical stage				
I &II	352 (40.8)	188(43.6)	116(44.1)	656 (42.3)
III & IV	510 (59.2)	243(56.4)	147(55.9)	900 (57.8)
Receiving ART				
Yes	750(86.9)	338(78.1)	216(80.0)	1304(83.3)
No	113(13.1)	95 (21.9)	54(20.0)	262(16.7)
Highest education attained				
Primary school and less	412(47.8)	207(47.9)	139(51.5)	758(48.5)
Secondary level	377(43.7)	179(41.4)	99(36.7)	655(41.9)
Tertiary level	73(8.5)	46(10.7)	32(11.9)	151(9.7)
Religious Affiliation				
Catholics	256(29.8)	212(49.0)	119(44.6)	587(37.6)
Anglican	272(31.6)	178(41.1)	122(45.7)	572(36.7)
Others	332(38.6)	43(9.9)	26(9.7)	401(25.7)
Employment status				
Yes	761(89.4)	384(88.9)	240(89.6)	1385(89.3)
No	90(10.6)	48(11.1)	28(10.5)	166(10.7)
Marital Status				
Single & never married	128(14.8)	49(11.3)	32(12.0)	209(13.4)
Married	393(45.5)	229(53.0)	139(52.1)	761(48.7)
Divorced/ widowed	342(39.6)	154(35.7)	96(36.0)	592(37.9)
Gender				
male	332(38.5)	208(48.0)	180(66.7)	720(46.0)
female	531(61.5)	225(52.0)	90(33.3)	846(54.0)
Self-reported medication adherence ≥ 95%				
Yes	749(86.8)	346(79.9)	180(66.7)	1275(81.4)
No	114(13.2)	87(20.1)	90(33.3)	291(18.6)

### Alcohol use outcomes at baseline

Overall, 703(44.9%) participants reported alcohol use within the last 6 months. Among the 703 alcohol users, the median (IQR) AUDIT score was 5 (2–10) points and 30 (4.3%) participants using alcohol had AUDIT scores > 19 points.

The majority of alcohol users, 433/703(61.6%) had AUDIT scores < 8 points and were categorized as non-hazardous drinkers (Table 1). A total of 863(55.1%) reported not using alcohol with in the 6 months preceding the enrolment interview. A total of 270/703 (38.4%) were taking alcohol at hazardous levels (AUDIT score ≥ 8), representing 17.2% of the whole study population. Participants with hazardous alcohol use were more likely to be males, with a shorter duration of HIV, and more likely to be non-adherent to medication (results not shown).

The three commonest alcoholic drinks reported were beer, reported by (89.2%) of alcohol users, gins/spirits (4.1%), *malwa*, a local maize based brew, (2.4%). Participants who consumed spirits also reported using other alcohol types. The median (IQR) units of alcohol consumed on a typical drinking day within the last month were 3(2–4) units among hazardous and 2(1–2) units among non- hazardous alcohol users, p < 0.0001. The median (IQR) number of drinking days within the last month were significantly higher among hazardous users compared to non-hazardous alcohol users 7 (2–13) days versus 1(0–2) days, p < 0.0001.

Overall, cigarette smoking was reported by 68 (4.3%) participants, of whom 20 (7.4%) were hazardous alcohol users, 28(6.4%) were non-hazardous alcohol users, while 20(2.3%) were not using alcohol.

In total, 1361 participants had at least one follow-up CD4+ T-cell count within the subsequent 12 months, giving a total of 2381 person visits of unique follow up CD4+ T-cell counts. The median (IQR) number of follow-up CD4+ T-cell counts was 2(1–2) per subject and was not different by alcohol use status i.e 2 (1–2), 2 (1–2), & 2 (1–2), p = 0.95 among the hazardous, non-hazardous and non-alcohol users respectively.

### Baseline immunological parameters

At enrollment, CD4+ T-cell counts tests done within a month of the interview was available for 1423 (90.9%) participants. The overall median (IQR) enrollment CD4+ T-cell count was 356 (243–516) cells/μl.

Among the non-ART participants, there was no statistically significant difference in the median (IQR) CD4+ T-cell count among participants with hazardous, non- hazardous alcohol use and non-alcohol use, 461(380–628)) vs. 503(405–682) vs. 534(399–677) cells/μl respectively, (p-value = 0.43) (Table 2). The median baseline CD4+ T-cell counts among participants on ART were not significantly different (p = 0.76) by alcohol use strata, i.e. 341.5 (247.5–462), 352(232–495) and 341(241–484) cells/μl among hazardous, non- hazardous and non-alcohol users respectively. Similarly, there was no difference in the median CD4+ T-cell percent by alcohol use strata i.e 19(17–25) vs. 24(20–27) vs. 23(16–28), p = 0.5 among non-ART participants and 16(11–23) vs. 17(12–21) vs. 16(11–23), p = 0.96 among on-ART participants between hazardous, non-hazardous, and non-alcohol users respectively.

**Table 2:** Comparison of median and interquartile range of CD4+ T-cell count and CD4 percentage by reported alcohol use category at baseline (n = 1423) and at all follow up visits (n = 1361) of PLHIV enrolled in the alcohol study at the Infectious Diseases Institute, Kampala Uganda.

PARTICIPANTS NOT ON ART					PARTICIPANTS ON ART			
Outcome	No alcohol use	Non-hazardous alcohol use	Hazardous alcohol use	Kruskal wallis P-value	No alcohol use	Non-hazardous alcohol use	Hazardous alcohol use	Kruskalwallis P-value
Baseline CD4+ T-cell count	534(399–677)	461(380–628)	503(405–682)	0.43	341(241–484)	341 (247.5–462)	352 (232–495)	0.77
Baseline CD4+ T-cell percent	23(16–28)	24(20–27)	19(17–25)	0.50	16(11–23)	17(12–21)	16(11–23)	0.96
Follow up CD4+ T-cell count	434(340–579)	459(386–601)	455(370.5–577)	0.30	321(226–491)	328(206–450)	348(230–499)	0.22
Follow up CD4+ T-cell percent	21(15–28)	21(17–27)	19(13.5–26)	0.35	14(9–23)	13(8–20)	13(9–20)	0.53

### Follow up immunological parameters

The median CD4+ T-cell count and CD4 percent at baseline and all follow-up visits lumped together are presented in Table 2, showing no significant differences between the median CD4+ T-cell count and CD4 percent in the three alcohol use categories.

In the univariable LME model among non-ART participants, there was no statistically significant difference in the mean change CD4+ T-cell count over 12 months of hazardous (estimate = 42.04, p = 0.11), and non-hazardous alcohol users(estimate = 29.84, p = 0.30) compared to non-alcohol users (data not shown). The initial multivariable model included CD4+ T-cell count at enrollment, sex, cigarette smoking, co-trimoxazole adherence level, CESD category, duration since testing HIV positive and the WHO HIV/AIDS clinical diseases stage.

In the final multivariable LME model (Table 3), among the non-ART receiving participants over the 12 months interval, the mean difference in theCD4+ T-cell count was -5.44 cell/μl, (p = 0.81) and -16.71cell/μl (p = 0.41) among non-hazardous and hazardous alcohol users respectively, compared to non-alcohol users controlling for baseline CD4+ T-cell count, time since enrollment, sex and WHO HIV/AIDS clinical stage.

**Table 3:** Final adjusted linear mixed effects regression of adjusted mean CD4+ T-cell count square-root andCD4+ T-cell percent among of participants with non-hazardous and hazardous alcohol use compared with non-alcohol users, stratified by ART receipt at the Infectious diseases Institute, Kampala Uganda.

Factor / Variable	NOT on ART				On ART			
	Adjusted estimate^1^ of mean CD4+ T-cell count (SE)	P-Value	Adjusted estimate^2^ of mean CD4+ T-cell percent (SE)	P-Value	Adjusted estimate^3^ of mean CD4+ T-cell count (SE)	P-Value	Adjusted estimate^4^of mean CD4+ T-cell percent (SE)	P-Value
Alcohol consumption category								
*Not using alcohol/Abstinent*	reference		reference		reference		reference	
*Non-hazardous alcohol use*	-5.44(21.90)	0.804	0.165(1.35)	0.90	6.22(7.03)	0.376	0.808(0.351)	0.20
*Hazardous alcohol use*	-16.71(20.24)	0.409	0.547(1.28)	0.67	4.83(6.09)	0.427	-0.267(0.334)	0.42
Time since enrollment (Days)	0.104 (0.042)	0.012	0.002(0.002)	0.41	-0.09(0.016)	<0.01	-0.007(0.002)	<0.01

^1^ n = 619 observations derived from 254 participants. Additional variables adjusted for in model but not shown: sex, WHO/HIV AIDS disease stage and baseline CD4+ T-cell count.

^2^n = 398 observations derived from 187 participants. Additional variables adjusted for in model but not shown: sex, WHO/HIV AIDS disease stage and baseline CD4 percent.

^3^ n = 2593 observations derived from 1026 participants. Additional variables adjusted for in model but not shown: Adherence level, CESD depression category, baseline CD4+ T-cell count and duration lived with HIV.

^4^ n = 1177 observations derived from 246 participants. Additional variables adjusted for in model but not shown: Adherence level and baseline CD4+ T-cell percent.

In the analysis among participants receiving ART, the univariable LME model, the mean difference in the CD4+ T-cell count was not different in hazardous alcohol use(estimate = 0.05, p = 0.99) and in the non-hazardous alcohol users (estimate = -13.2.33, p = 0.31) compared to non-alcohol users (data not shown). After adjusting for the baseline CD4+ T-cell count, self -reported adherence category, sex, CES-D depression score and duration of HIV disease, the mean difference in CD4+ T-cell count over the 12 months was 4.83 cells/μl, higher for hazardous alcohol users compared to non-alcohol users, (p = 0.43) and 6.22 cells/μl higher for non-hazardous alcohol users compared to non-alcohol users (P = 0.38).

Similarly, in the secondary analyses with CD4 percent as the outcome, the final LME models among participants on ART (Table 2),there was no statistically significant difference in the mean CD4+ T-cell percent over 12 months of participants not using alcohol and participants with hazardous alcohol use (mean CD4 percent difference = -0.27, p = 0.42) and with non-hazardous alcohol use (mean CD4 percent difference = 0.81, p = 0.205), controlling for self- reported ART adherence, WHO HIV/AIDS clinical diseases stage, duration of HIV positivity and sex. Among non-ART receiving participants, CD4 percent over 12 months was not different among both hazardous (mean CD4 percent = 0.55, p = 0.67), non- hazardous alcohol users (mean CD4 percent difference = 0.176, p = 0.90) compared with non-alcohol users. Of note is that males had a statistically lower CD4 percentage than females in models of non-ART (mean difference in CD4 percent estimate = -4.8, p = 0.001) and ART receiving participants (estimate = -3.6, p = 0.001).

In order to test whether any effect of alcohol use on CD4+ T-cell count among persons on ART is mediated through ART non-adherence, we initially tested the association between alcohol use and non-adherence to ART (< 95% ART adherence). We found a linear increase in the risk of non-adherence to ART of [0.38(0.26–0.50), p < 0.001] with each level increase category of alcohol use from non-alcohol use, to non-hazardous and then to hazardous alcohol use adjusting for age, gender, duration lived with HIV and visit interval since enrolment.

From the structural equation modeling analysis, there was no statistically significant total effect of alcohol use category on CD4^+^ T-cell count 0.46 cells/μl, 95% CI [-2.65, 3.58],p = 0.77. On decomposing the latter effect, the natural direct effect of alcohol use on CD4^+^ T-cell count was not statistically significant, 1.37 cells 95%CI [-1.78, 4.52], p = 0.39, however there was a statistically significant natural indirect effect of alcohol use on reduction of CD4^+^ T-cell count (-0.91 cells/μl [-1.36, -0.45], p < 0.001) that was mediated through ART non-adherence.

## Discussion

Among HIV infected men and women in an HIV clinic in urban setting in Uganda, self -reported alcohol consumption within the past 6 months was not associated with lower CD4+ T-cell counts at assessment and over the next 12 months in both ART receiving and non-ART receiving participants.

The main difference between studies that have explored this association is the difference in the mode of assessment of the exposure (alcohol use) and the outcome (CD4+ T-cell count). For example, *Samet et al* assessed alcohol use by CAGE questionnaire and the outcome was the average difference in CD4+ T-cell count between abstinent participants and heavy/moderate alcohol use over a 7 year period. *Chandiwana et al* looked at any alcohol consumption and CD4+ T-cell count six months later as the outcome[11,13]. Unlike previously published studies, our study assessed alcohol use using the AUDIT which is an effective tool for identifying participants with hazardous drinking. One advantage of the AUDIT is its ability to provide a standard way to measure alcohol consumption which allows for future cross- study comparisons[29].

The effect of alcohol on CD4+ T-cell count among persons on ART may not be easily elicited because of the overwhelming impact of ART on CD4+ T-cell count, which may obscure any of the effects of alcohol if they indeed exist and therefore finding a critical sample of persons taking alcohol in amounts that affect CD4+ T-cell count may require restricting the study to participants taking the highest amounts of alcohol. Advanced statistical techniques such as marginal structural models may elicit the direct contribution of alcohol to the poor immunological response[30,31]. Unlike *Kahler et al* who demonstrated that alcohol had both a natural direct and natural indirect effect on CD4+ T cell count among a cohort of cohort of patients receiving care for HIV, we did not observe any direct effect of alcohol on CD4+ T cell count in our study[32]

The impact of alcohol on CD4+ T-cell count may be demonstrated through the behavioral effect of alcohol use in moderating adherence to ART resulting into impaired immunological and virological response to ART We demonstrated that alcohol users receiving ART had lower self-reported adherence levels compared to non-alcohol users and further demonstrated that this lower ART adherence level among alcohol users was significantly associated with lower CD4 cell counts implying that there was a statistically significant natural indirect effect of alcohol use on reduction of CD4^+^ T-cell count that was mediated through ART non-adherence.

Many PLHIV skip or forget ART medication while drinking alcohol [33] or consciously decide to not mix alcohol with ART medication[34]. The level of non-adherence that would affect immunological parameters and the duration through which any CD4+ T-cell count decline would manifest are unknown. Given that current ART combinations still provide good virological suppression and immunological response at adherence levels slightly lower than 95%, it is possible that the non-adherence to ART in the majority of patients was not too low to result into reduced CD4+ T-cell count response [35]. Additionally the 12 months of follow-up in our study may not be long enough to demonstrate any immunological effects if they indeed exist.

Another possible reason for lack of demonstrable effect of hazardous alcohol use on CD4+ T-cell count was the very few (4.1%) participants reporting consuming liquor beverage type. It has been postulated that alcohol beverage type, specifically liquor spirits may reduce the CD4+ T-cell count through reducing the participants’ thymus size, which is responsible for de novo generation of new T cells [36].

Among the limitations in this study is the 12 months of study follow-up. A longer follow-up duration may be needed to elicit any direct alcohol related immunological changes. Another limitation is the use of baseline alcohol data making the likelihood of hazardous alcohol use misclassification during follow-up likely hence obscuring the association we were testing. Assessment of alcohol use by self-report may underreport alcohol use [37], but this underreport would affect the classification if only the heaviest drinkers misreported themselves as light or nondrinkers altogether. Similarly, it is possible that the self-reported medication adherence was also under-reported as a manifestation of social desirability bias. Although we have no way of mitigating these altogether, assurances of privacy and confidentiality, the use of non-clinical staff in alcohol interviews may have minimized the under-report in these socially desirable behavior. We found participants at different stages of their HIV disease progression which may naturally have an impact on their CD4 trajectories, but we adjusted for the HIV duration since testing HIV positive in multivariable analyses.

### Clinical implications

Among participants with a low CD4+ T-cell count and concurrent alcohol use, other potential causes of a low CD4+ T-cell count in general still need to be excluded including potential harmful behaviors including missing ART [22]. Furthermore, during routine clinical visits, participants reporting alcohol use should have a detailed exploration of adherence to ART, irrespective of CD4 cell count or CD4 cell count trajectory. Where possible, regimen choice for participants with reported alcohol use should consist of fewer dosing frequencies, preferably once a day dosing, that can be scheduled at the most convenient time to not be influenced by alcohol use so that, participants may plan *apriori* on the dosing of their medication prior to drinking alcohol.

With the current recommendation of treating all HIV infected persons irrespective of CD4+ T-cell count level, future studies should explore the impact of alcohol on both CD4+ T-cell count and plasma viral load and in addition collect data on alcohol beverage type to test the potential impact of alcohol beverage type on immunological and virological response in this setting.

### Conclusions

Hazardous alcohol use, among men and women with HIV attending an urban clinic in Kampala Uganda was not associated with lower CD4+ T-cell count. However, among ART receiving PLHIV, we observed statistically significant CD4+ T-cell count reductions mediated through alcohol induced ART non-adherence.

Among PLHIV with lower than expected CD4+ T-cell count, alcohol consumption should be excluded as an underlying factor for non-adherence to ART and any interventions targeting alcohol use should tackle possible ART non-adherence.
